# Diagnostic Testing for Degenerative Disc Disease

**DOI:** 10.1155/2012/413913

**Published:** 2012-07-12

**Authors:** Michael W. Hasz

**Affiliations:** Virginia Spine Institute, 1831 Wiehle Avenue, 2d Floor, Reston, VA 20190, USA

## Abstract

The diagnostic of degenerative disc disease should be reached with the help of various diagnostic studies. This article briefly review the information gained by the following tests: radiographs, computed tomography, magnetic resonance, and discography. The article explains how each modality provides a piece of the diagnostic puzzle and how discography confirms the origin of the patient's pain.

## 1. Introduction

In the diagnosis and treatment of a patient with ongoing predominantly midline low-back pain (axial back pain), degenerative disc disease must be kept high amongst the possible diagnoses. In addition to the appropriate patient history, examination, and patient response to nonoperative conservative treatment, various diagnostic studies can aid in the diagnosis of degenerative disc disease and the exclusion of other diagnoses.

Common studies used to aid in the diagnosis of patients with axial back pain include lumbar radiographs, computed tomography (CT) scan, magnetic resonance imaging (MRI), and provocative discography. These studies should be used in conjunction with the patient history and physical examination. They are useful to aid in the diagnosis but are not in and of themselves definitive studies for the diagnosis of pain. However, using the studies in conjunction with the patient's clinical status and response to treatment is very useful for the overall diagnosis of degenerative disc disease.

## 2. Lumbar Radiographs

Lumbar X-rays should include a full series with standing or weight-bearing views: standing anterior-posterior (AP) pelvis and lateral flexion-extension views. These weight-bearing and dynamic studies can help identify many diagnoses which may otherwise be overlooked by a pure supine or non-weight-bearing X-ray: instability, increased angular motion on flexion-extension lateral views, anterolisthesis or retrolisthesis (each of which can be either subtle or direct indications of local instability), or indirect findings of lumbar disc degeneration.

**Figure 1:**
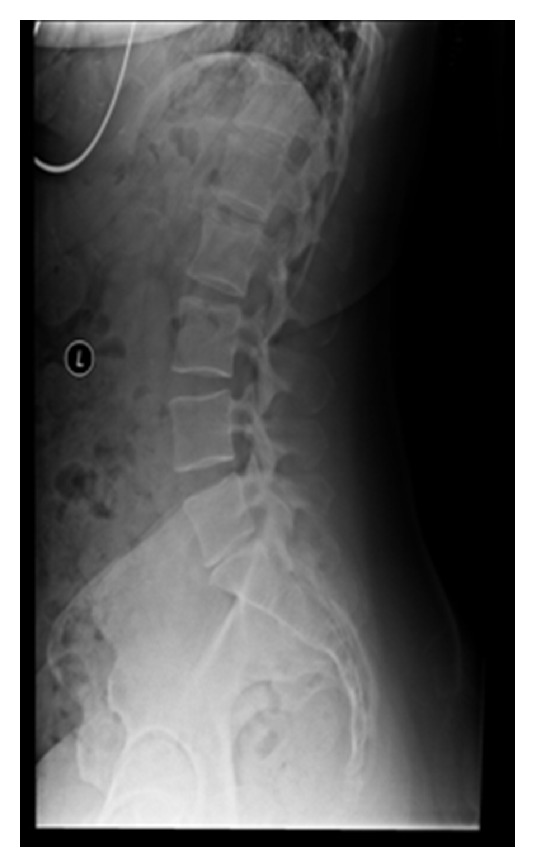
A lumbar radiograph with narrowing of the L5–S1 disc space. This narrowing is suggestive of disc degeneration.

Radiographs are more often used to exclude other diagnoses rather than directly diagnose degenerative disc disease. Diagnoses that can be more directly excluded with appropriate X-rays include scoliosis, spondylolisthesis, fractures, and gross instability. The actual radiographic findings of lumbar disc disease encompass a range of findings used to infer disc disease (Figure 1).

**Figure 2:**
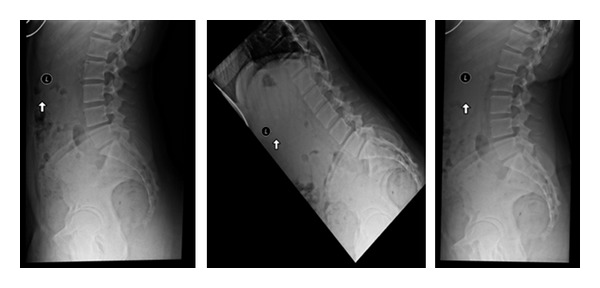
Lateral standing, flexion and extension X-rays are important to identify motion and/or instability in the spine. Some patients have unremarkable lateral upright X-rays but demonstrate a mobile spondylolisthesis upon dynamic testing.

The radiographs are primarily used for assessing bony anatomy and alignment. They do not directly view the discs and soft tissues. In the early stages of lumbar disc disease, the disc heights may be unchanged. There may be annular tears identified and painful discs, but radiographs may not give any significant indication of disc injury, particularly in the acute setting of a disc injury. The flexion-extension lateral views may hint to muscle spasm and decreased excursion of range of motion. Therefore, muscle spasm or restriction can be inferred but not directly attributed to disc disease (Figure 2).

**Figure 3:**
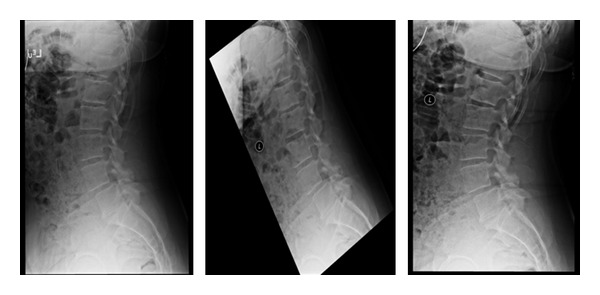
Lateral X-rays demonstrating a L4-L5 grade 1 spondylolisthesis. The standing lateral dynamic flexion and extension X-rays are used to determine motion at this segment.

Some patients may have instability related to insufficiency of the lumbar disc. Some authors have defined 11° or greater of angular change on flexion-extension views to suggest the disc to be unstable [1]. Additionally lumbar retrolisthesis identified on radiographs has also been used to infer instability at lumbar levels [2] (Figure 3).

The angular changes as well as retrolisthesis in the degenerative model of disc disease should not be confused or associated with the trauma model associated with White and Panjabi studies [1]. These studies were performed on cadavers where acute injury models and structural defects were made in order to assess instability in a trauma model. Applying these criteria in the degenerative disc disease model would be inappropriate.

**Figure 4:**
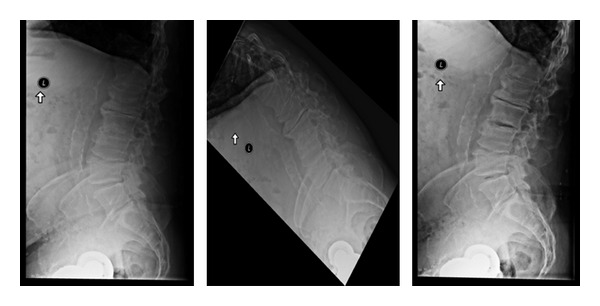
Lumbar X-rays. Standing lateral, flexion, and extension views. Disc degeneration suggested by vacuum disc (on extension) and osteophyte formation.

Further suggestion of degenerative disc disease along the degenerative cascade would lead toward the formation of osteophytes along the edges of the endplates, narrowing of the disc space height, increased sclerosis along the endplates at the disc segment level, and possible osteophytes or sclerosis of the facet joints. In conjunction with the loss of disc space height, the foramina can be observed to narrow on the lateral and oblique studies [3] Toward the end of the degenerative cascade, a vacuum disc element can frequently be observed [4] (Figure 4). 

Many of these degenerative changes in the lumbar spine may not be symptomatic and are only suggestive of the diagnosis of degenerative disc disease. In symptomatic patients, these radiographic findings are definitely suggestive of degenerative disc disease although further studies would be indicated. 

## 3. Computed Tomography Scan

Overall a CT scan by itself is of limited value in the correct diagnosis of degenerative disc disease. Often a CT scan can be normal in the face of this disease. A CT scan has little direct value beyond the lumbar radiographs in the direct assessment of degenerative disc disease.

A CT scan is used to help exclude other diagnoses, as previously mentioned on the section on plain radiographs. A CT scan is very useful to help assess a pars defect or spondylolisthesis for example. Additionally, a CT scan can demonstrate findings that are also found on radiographs as well. A CT scan may be able to better demonstrate osteophytes as well as endplate sclerosis and vacuum disc sign, all related to findings of degenerative disc disease [4]. 

**Figure 5:**
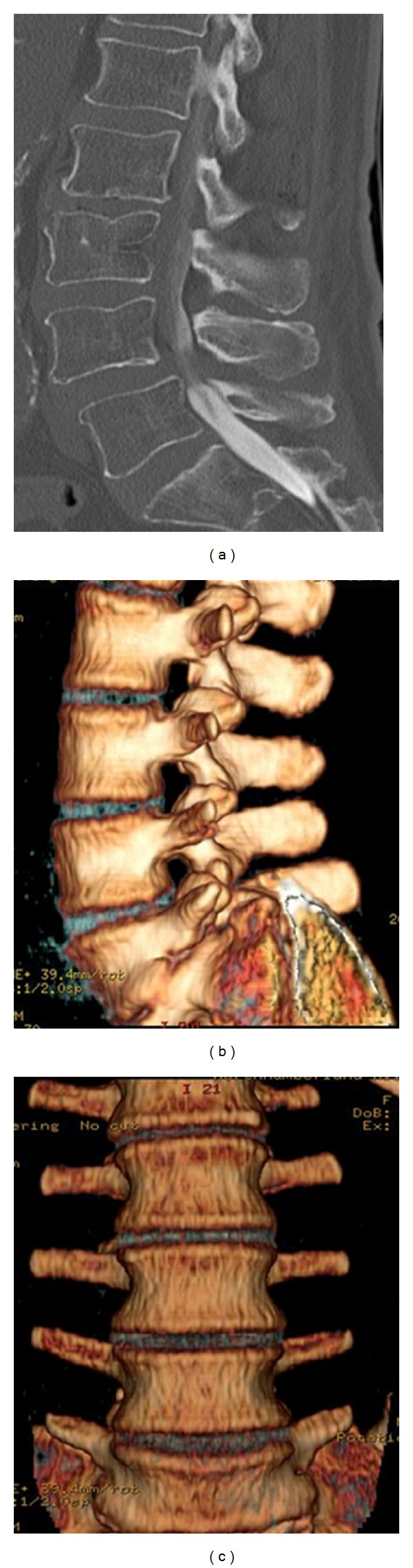
CT scans. (a) Grade I spondylolisthesis. ((b) and (c)) 3D reconstruction, lateral and anterior views.

A CT scan is performed in a non-weight-bearing position and is of limited use to assess any dynamic instability in the lumbar spine. The CT scan can be used to assess the spinal canal and vertebral bony anatomy, as well as the posterior joint complex. It can further assess potential foraminal stenosis, and, when used in conjunction with myelography, it can assess possible nerve compression and indirectly disc protrusions (Figure 5).

## 4. Magnetic Resonance Imaging

MRI scanning, like CT scanning, can be used to evaluate the spinal canal and space available for neural structures. It can evaluate the overall bony alignment and the lumbar facets, but it has the additional benefit of allowing the direct assessment of the neural structures as well as the disc structures. This direct evaluation of neural and disc structures is not possible by CT scan [5].

**Figure 6:**
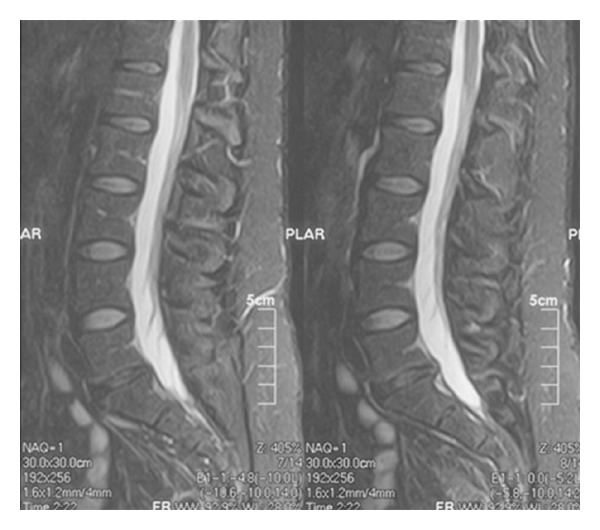
MRI sagittal T2-weighted demonstrating decreased disc signal at L5-S1. This indicates decreased hydration of the disc space. It does include or exclude patient symptoms of back pain.

An MRI is capable of evaluating the hydration within the discs based on increased signal on the T2-weighted images. Increased disc signal on T2-weighted images is associated with dehydration of the lumbar discs. Change in the disc signal, or darkening of the signal, is associated with dehydration or loss of hydrogen ions within the disc. This is often associated with lumbar disc degeneration. Decreased hydration leads to a loss of signal intensity on the T2 images which leads to darkening of the disc on the image (Figure 6). An area of increased signal may be identified within the disc or along the annulus of the disc. This area of increased signal is called a high-intensity zone. This high-intensity zone is thought to be correlated with an area of increased inflammation, thought to be associated with a disc tear or annular tear, and often is associated with axial back pain [6].

In addition to the changes within the disc, changes at the endplate adjacent to the disc have been described [7]. Modic noted reactive endplate changes at the endplates of the discs and graded them as Modic 1, Modic 2, and Modic 3 changes.

**Figure 7:**
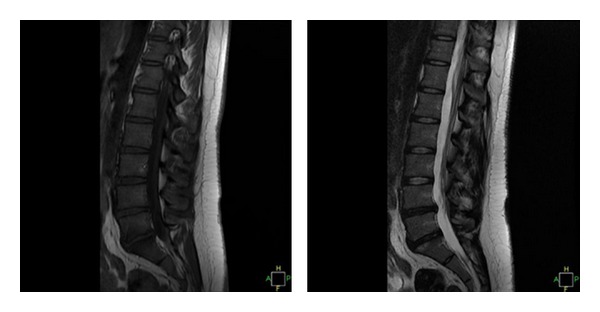
This MRI has two levels of decreased disc signal: L4-5 and L5-S1. There are early reactive endplate changes at L5-S1 as well (Modic type 1).

- Modic 1 reactive endplate changes demonstrate decreased disc signal on T1 images and increased disc signal on T2 images. These changes are associated with disruption and fissuring of the endplate and vascular fibrous tissue adjacent to the endplate. Modic 1 reactive endplate changes are infrequently associated with axial back pain (Figure 7).
- Modic 2 reactive endplate changes are represented by increased signal intensity on T1 images and neutral signal on T2 images. These changes are associated with degenerative disc changes on plain radiographs. These changes represent yellow marrow replacement in the adjacent vertebral body at the endplates. This increased lipid content has been suggested to be an inflammatory response associated with a painful disc.
- Modic 3 reactive endplate changes demonstrate decreased disc signal on both T1 and T2 images. This represents bony sclerosis at each endplate. There are sclerotic endplate changes representing near-end-stage disease at the endplates. These are also associated with decreased blood supply at the endplates.

As mentioned earlier, the discs themselves have a range of intensity from high signal on the sagittal T2 images to a loss of signal. This represents a range from the normal hydration of the disc to gradual loss of hydration which is represented by a breakdown of the proteoglycans within the disc space and gradual degeneration of the disc.

A dark disc on MRI does not necessarily mean it is a symptomatic disc. Disc abnormalities are frequently seen on MRI in an asymptomatic patient. Up to 30% of asymptomatic volunteers have an approximately 30% rate of abnormal signal intensity within the discs. These abnormalities include disc protrusions and herniations, as well as decreased disc signal. Additionally as a patient ages, the frequency of decreased disc signal on MRI increases.

However, in a clinically symptomatic patient, an MRI that demonstrates decreased disc signal, particularly along the posterior annulus known as a high intensity zone, is highly associated with axial back pain. In symptomatic patients, lumbar discography can be of further use to help determine whether or not a disc is symptomatic.

In summary, an MRI plays an important but not exclusive role in the diagnosis of degenerative disc disease. In a symptomatic patient who has failed nonoperative conservative treatment and has normal X-ray findings, an MRI can be a very useful tool for further evaluation of a patient with axial back pain. A dark disc can be a tool to diagnosis of symptomatic degenerative disc disease.

## 5. Discography

**Figure 8:**
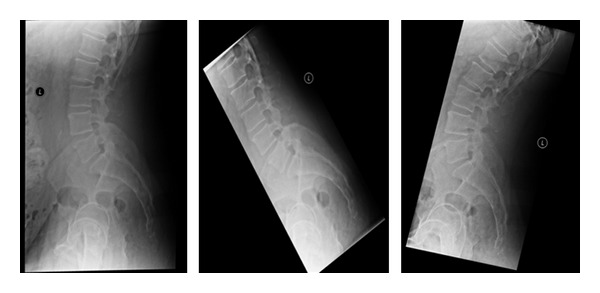
This patient's X-rays, including the upright lateral flexion and extension, are overall unremarkable.

**Figure 9:**
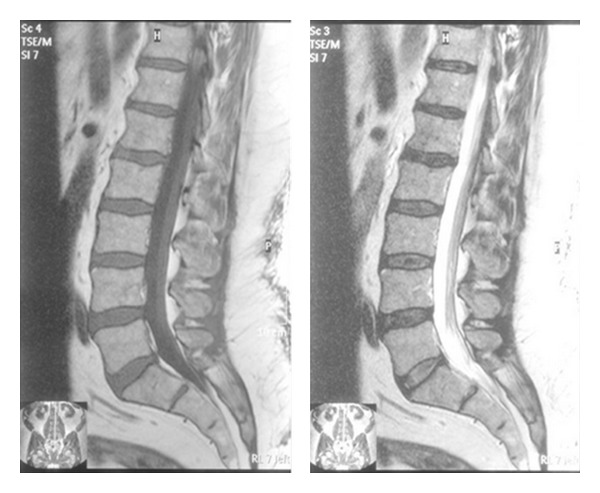
The same patient's T1 and T2 MRI demonstrates decreased disc signal at L4-5.

Discography, particularly provocative discography, is the single most important diagnostic tool of degenerative disc disease. Lumbar discography is a test that would be appropriately performed in symptomatic patients who have failed nonoperative conservative treatment and whose X-rays and MRI studies suggest no other obvious pathology leading toward their diagnosis (Figures 8 and 9). Since the patient's chief complaint is pain and since no imaging studies actually see pain, lumbar discography can be used to potentially provoke and reproduce the patient pain [8].

There are four important pieces of information obtained in an appropriately performed discography: the subjective pain response, the volume and/or pressure of the fluid injected into the disc (a normal disc accepts 0.5 to 2.5 cc), the morphology of the disc injected (http://www.ncpainmanagement.com/InfoLumbarDiscography.htm), and the lack of a pain response in the adjacent controlled disc levels tested. All four of these criteria can be used and evaluated in an appropriately performed discography.

**Figure 10:**
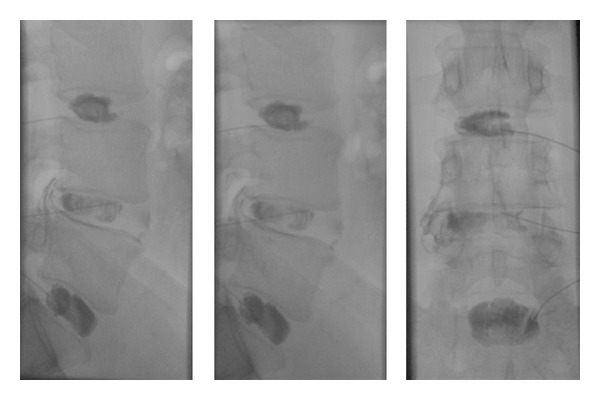
The discography performed on the same patient demonstrated tears at L4-5 and reproduced the patient's pain. The other levels were pain free and morphologically normal.

The discography should be performed under fluoroscopic guidance and using standard aseptic technique. Fluoroscopic guidance is used and radiopaque dye is injected within the disc space. This contrast enables the imaging of the actual disc, and its morphology can be evaluated. A normal disc has a biloped or globular pattern within the center of the disc. An abnormal pattern demonstrates leakage of the dye to the various layers of the annulus and possible leakage into the epidural space (Figure 10). Additionally multiple discs need to be tested in order to identify lack of pain response in adjacent disc levels tested [9].

The most important portion of the discography is the actual pain response of the patient. It is important to identify discs adjacent to the symptomatic level to be painfree or minimal pressure sensation. The quality of the pain should be reported by the patient using self-reported pain intensity visual analog scale. Observation of the patient by the discographer can also determine the patient's pain response and behaviors. The patient needs to be alert and cooperative for the procedure in order to monitor these responses.

Postdiscography CT scan has been reported by some to increase the ability to diagnose radial tears in the annulus. Based on current treatment options of degenerative disc disease, including lumbar fusion and/or prosthetic disc placement, this additional information may not be of any clinical significance. The specificity and location of the annular tear may become useful information for future treatment options.

In summary, the provocative discography evaluation is the only test currently available to evaluate the actual pain response of a patient, as other imaging studies such as CT scan, X-rays, and MRI can only infer anatomic changes and cannot evaluate pain directly. It is useful to identify levels of degenerative disc disease that recreate the patient's pain. It is significantly useful to identify levels adjacent that do not recreate their pain.

## 6. Conclusion

When evaluating a patient with ongoing axial back pain with predominantly back pain as opposed to radicular pain, many studies such as X-rays and an MRI are the initial imaging studies to obtain. If more specific information is needed, lumbar discography is the most direct study available to further evaluate pain with the diagnosis of degenerative disc disease.
